# Effect of *Cotesia ruficrus* Parasitization on Diversity and Community Composition of Intestinal Bacteria in *Spodoptera frugiperda*

**DOI:** 10.3390/insects15080570

**Published:** 2024-07-27

**Authors:** Xian Li, Jing-Jing Jia, Jun-Long An, Fan-Xin Meng, Tong-Xian Liu, Shi-Ze Zhang

**Affiliations:** 1 State Key Laboratory of Crop Stress Biology for Arid Areas, Key Laboratory of Plant Protection Resources and Pest Management of Ministry of Education, Key Laboratory of Integrated Pest Management on Crops in Northwestern Loess Plateau of Ministry of Agriculture and Rural Affairs, College of Plant Protection, Northwest A&F University, Yangling 712100, China; lixian0728@126.com (X.L.); anjunlong@nwafu.edu.cn (J.-L.A.); mengfanxin@nwafu.edu.cn (F.-X.M.); 2 Institute of Plant Protection, Hainan Academy of Agricultural Sciences, Research Center of Quality Safety and Standards for Agro-Products, Hainan Academy of Agricultural Sciences, Haikou 571100, China; j9405136318@163.com; 3 Institute of Entomology, College of Agriculture, Guizhou University, Guiyang 550025, China; tx.liu@gzu.edu.cn

**Keywords:** *Cotesia ruficrus*, fall armyworm, gut microbiota, abundance, 16S rRNA

## Abstract

**Simple Summary:**

*Cotesia ruficrus* (Haliday) (Hymenoptera: Braconidae) is a native enemy of the important invasive fall armyworm pest, *Spodoptera frugiperda* (J.E. Smith) (Lepidoptera: Noctuidae), in China, exhibiting significant pest control capabilities. To investigate the impact of *Cotesia ruficrus* on the gut bacteria of fall armyworm caterpillars following parasitism, we used 16S rRNA sequencing technology to analyze the diversity and richness of gut bacteria in both long-term and short-term laboratory fall armyworm caterpillars. Our study reveals that *Cotesia ruficrus* parasitism induces changes in fall armyworm larval gut microbiota, thereby shedding light on the capacity of parasitoid parasitism to impact host gut microbiota. These findings lay the foundation for deeper exploration of the gut microbiota’s role in the parasitoid–host interaction mechanism.

**Abstract:**

Parasitoids have the potential to alter the gut microbiota of their host insects post-parasitization, thereby influencing the host’s physiological functions and creating a more favorable environment for the survival of the parasitoid’s progeny. *Cotesia ruficrus* is a native enemy of the important invasive fall armyworm (FAW) pest, *Spodoptera frugiperda*, in China, exhibiting significant pest control capabilities. To investigate the impact of *C. ruficrus* on the gut bacteria of FAW caterpillars following parasitism, we used 16S rRNA sequencing technology to analyze the diversity and richness of gut bacteria in both long-term laboratory and short-term laboratory FAW caterpillars. The results revealed *Enterococcus* as the predominant bacteria across all treatments, while no significant differences were observed in the diversity and richness of gut bacteria between non-parasitized and parasitized long-term laboratory FAW caterpillars. Similarly, while the diversity of gut bacteria in non-parasitized and parasitized short-term laboratory FAWs showed no significant variance, a marked discrepancy in richness was noted. Moreover, the richness of gut bacteria in short-term laboratory FAW caterpillars surpassed that of their long-term laboratory counterparts. In addition, it was found that *Corynebacterium* existed only in the intestinal tract of FAW caterpillars that were parasitized by *C. ruficrus*. These results substantiate that *C. ruficrus* parasitization can alter the gut microbiota of FAW caterpillars, providing valuable insights into the interplay between gut microbiota and the dynamics of parasitoid–host interactions.

## 1. Introduction

The insect gut harbors a diverse array of symbiotic microorganisms, including bacteria, fungi, and viruses, with bacteria being the most prevalent [1]. Insects create a conducive environment and nutrient supply for these microorganisms [2], which play crucial roles in various aspects of insect biology such as reproduction, growth, development, metabolism, immunity, behavior, and detoxification processes [3,4,5]. For example, honeybees with gut microbiota are heavier than those without them, suggesting that the gut microbiota of honeybees can increase the weight of the host [6]. Compared to *Drosophila melanogaster* Meigen (Diptera: Drosophilidae) with *Lactobacillus* and *Acetobacter*, *D. melanogaster* without bacteria have poorer learning and memory abilities [7]. Additionally, certain adult *Eretmocerus mundus* Mercet (Hymenoptera: Aphelinidae) harbor esterases-secreting *Arthrobacter* that aid in pesticide degradation [8]. Furthermore, the composition of insect gut microorganisms is affected by factors such as host developmental stage, diet, season, habitat, and exposure to pesticides. Notably, in fully metamorphic insects like Lepidoptera, gut microbial populations change with each molt [9]. For instance, the predominant gut microorganism in *Heliothis virescens* Fabricius (Lepidoptera: Noctuidae) shifts from Enterobacteriaceae when feeding on chickpeas and tobacco to Methylobacteriaceae when feeding on cotton [10].

The fall armyworm (FAW), *Spodoptera frugiperda* (J.E. Smith) (Lepidoptera: Noctuidae), originally hails from tropical and subtropical regions of the Americas [11]. Known for its strong migratory and reproductive capabilities, short life cycle, broad host range, and high pesticide resistance [12], the FAW has extended its presence to the African continent and has heavily impacted extensive regions across the Middle East, Asia, and the Pacific, thereby posing a severe threat to global food security [13]. The FAW first invaded China’s Yunnan Province in January 2019 and has since spread to 27 provinces, inflicting substantial losses on the country’s corn production [14]. Recent studies have shed light on the gut microbiota of the FAW, highlighting Proteobacteria and Firmicutes as the predominant bacterial phyla [15]. The gut microbial community of FAWs is intricately affected by various factors, showing distinct variations across different developmental stages and in response to varied host plants, with soybean-fed FAW caterpillars exhibiting higher microbial diversity than those fed on corn [16,17]. Moreover, laboratory-cultured FAWs display lower gut microbial richness and diversity compared to their field counterparts [16]. In addition, chemical pesticides also affect the gut microbiota of FAWs [18].

**Figure 1:**
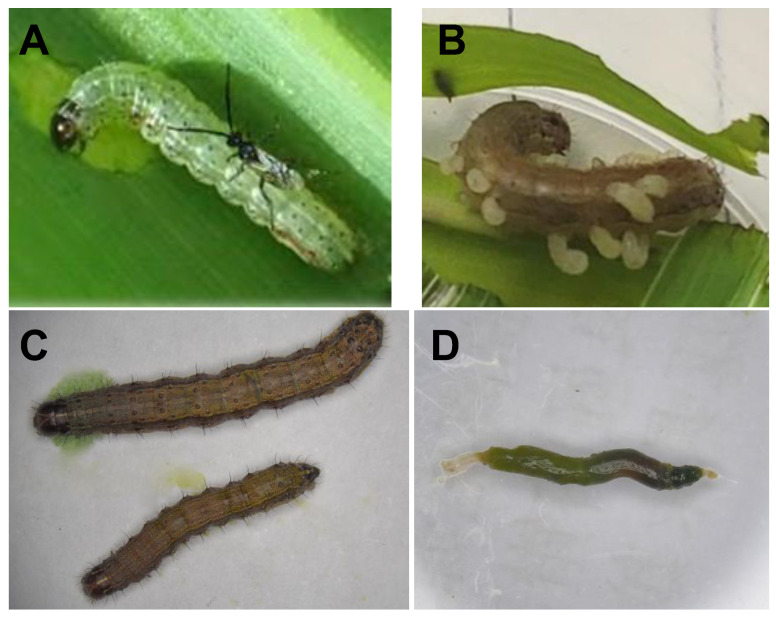
*Spodoptera frugiperda* larva and its gut. (**A**) *Cotesia ruficrus* is parasitizing *S. frugiperda*; (**B**) third instar larvae of *C. ruficrus* crawling out of *S. frugiperda* larva; (**C**) *S. frugiperda* larvae; (**D**) gut of *S. frugiperda* larva.

Recent reports in the literature have highlighted the ability of parasitoids to modulate the gut microbiota of their host insects post-parasitism [19,20,21,22]. In 2020, *Cotesia ruficrus* (Haliday) (Hymenoptera: Braconidae), a local parasitoid, was first observed parasitizing FAW caterpillars in Chinese corn fields. *C. ruficrus* is a gregarious endoparasitoid of the FAW, parasitizing larvae from the first to third instar. Its larval development within the FAW lasts approximately 8 to 10 days post-parasitization, after which it emerges from FAW caterpillars to pupate (Figure 1), ultimately resulting in the death of the FAW before it can pupate; this highlights its potential as an effective biological control agent for the invasive pest [23,24,25,26]. However, the effect of *C. ruficrus* parasitism on the gut microbiota of the FAW has not yet been reported. Here, 16S rRNA sequencing technology was used to analyze the effects on gut bacterial diversity and community composition in long-term laboratory and short-term laboratory feeding FAW populations. These findings lay the groundwork for further exploration into how *C. ruficrus* affects host–parasitoid interactions by modifying the gut microbiota of FAWs.

## 2. Materials and Methods

### 2.1. Insect Rearing

The long-term laboratory population of FAWs was procured from corn fields in the Yangling District of Xianyang (34°17′ N, 108°01′ E), China, in July 2019. These specimens were subsequently reared in the laboratory with the *Zea mays* L. var. Shandan 636 for a long time to establish a laboratory population. By contrast, the short-term laboratory population of FAWs was obtained from corn fields in the Xiuying District of Haikou (20°02′ N, 110°28′ E), China, in November 2023. The collected FAW specimens were reared in the laboratory on corn leaves until they pupated, and the emerged adults mated and laid eggs. These eggs were subsequently collected and incubated, and the resulting larvae were fed on corn leaves until they reached the third instar stage (3L), which was used for the experiments. For sex discrimination of the FAWs, the method outlined by Visser was followed [27].

Concurrently, a population of *C. ruficrus*, a parasitoid of FAW caterpillars, was collected in July 2020 from parasitized FAW caterpillars in the corn fields of the Zhouzhi District in Xi’an (34°09′ N, 108°13′ E), China. The parasitized FAW caterpillars were introduced into the laboratory and reared on corn leaves. Upon the emergence of parasitoids, they were then provided with first to third instar FAW larvae as hosts. All experimental insect populations were maintained within an intelligent artificial climate chamber (Shanghai Yiheng Scientific Instrument, Shanghai, China) under controlled conditions, including a temperature of 26 ± 1 °C, relative humidity (RH) of 70% ± 5%, a photoperiod of 14 h light and 10 h darkness (14L:10D), and supplemented nutrition comprising 10% honey water.

### 2.2. Gut Collection

The 3rd-instar long-term laboratory FAW caterpillars were individually placed in a test tube with a diameter of 1.2 cm and a length of 6.0 cm. Subsequently, a mated female parasitoid (1-day-old) was introduced into the test tube. The opening of the tube was sealed with absorbent cotton, and upon observation of parasitism, the host larvae were transferred and placed in a rearing jar (diameter 3 cm, height 4.0 cm) with fresh corn leaves for individual cultivation. Once the FAW caterpillars reached the 5th instar stage, they were subjected to a sequential treatment involving immersion in 0.5% NaClO for 2 min, followed by 75% ethanol for 1 min, and ultimately rinsed thrice with sterilized–deionized water. Following this, ten gut samples from the FAW caterpillars were dissected under a dissecting microscope (JSZ6, Jiangnan Yongxin Optical Co., Ltd., Nanjing, China) in a 0.01 M PBS buffer solution (pH = 7.4) and were collected in a 1.5 mL centrifuge tube. These samples were quickly frozen using liquid nitrogen and stored in a −80 °C refrigerator. Additionally, ten 5th-instar long-term laboratory FAW caterpillars that had not been parasitized were gathered to serve as a control group. The same collection method was applied to obtain gut samples from both parasitized and non-parasitized short-term laboratory FAW caterpillars. In total, four treatments were conducted, denoted as long-term laboratory parasitized (LP), long-term laboratory non-parasitized (LNP), short-term laboratory parasitized (FP), and short-term laboratory non-parasitized (FNP), with each treatment being replicated three times.

### 2.3. DNA Extraction and 16S rRNA Sequencing

The total DNA extracted from the gut samples was obtained according to the instructions of the MasterPure Complete DNA and RNA Purification Kit (Epicentre, Lindenhurst, IL, USA) and subsequently assessed for quality using the NanoDrop 2000 (Thermo Scientific, Waltham, MA, USA). Subsequently, the amplification of the V3-V4 region of 16S rRNA was performed using primer pairs 338F (5′-ACTCCTACGGGAGGCAGCAG-3′) and 806R (5′-GGACTACHVGGGTWTCTAAT-3′). The PCR amplification system comprised 4 μL of 5 × FastPfu Buffer, 2 μL of 2.5 mM dNTPs, 0.8 μL each of 5 μM forward and reverse primers, 0.4 μL of FastPfu Polymerase, 0.2 μL of BSA, 10 ng of template DNA, and double-distilled water (ddH_2_O) to reach a final volume of 20 μL. The PCR reaction program was executed as follows: initial pre-denaturation at 95 °C for 3 min, followed by 30 cycles of denaturation at 95 °C for 30 s, annealing at 50 °C for 30 s, and extension at 72 °C for 45 s, with a final extension step at 72 °C for 10 min. The sequencing of the gut samples was conducted by Shanghai Meiji Biomedical Technology Co., Ltd. Sequencing libraries were prepared following the instructions of the TruSeqTM DNA Sample Prep Kit (New England Biolabs, Ipswich, MA, USA) and sequenced using the Illumina MiSeq PE300 platform.

### 2.4. Sequence Data Analysis

The PE reads were assembled, and subsequent quality control and filtering steps were performed using the fastp version (0.19.6) for sequence quality assessment. Assembly of the double-end sequence was accomplished through FLASH (1.2.11). Operational taxonomic units (OTUs) were generated by clustering sequences with a 97% similarity threshold using Uparse (7.0.1090). Taxonomic classification annotation was performed utilizing the RDP classifier (2.1) with a comparison threshold set at 70%. Analysis of community structure at various taxonomic levels was possible based on taxonomic information. The normalization of data for each sample was achieved by constructing a dilution curve using the extracted sequence numbers and their corresponding OTU counts. Alpha diversity metrics, such as the Chao index, Ace index, Shannon index, and Simpson index, were calculated through Mothur (1.30.2). Principal Component Analysis (PCA) and Principal Coordinate Analysis (PCoA) were carried out based on Unweighted unifrac. A Student’s t-test was used to compare the Alpha diversity indices of gut bacteria in FAW caterpillars with and without parasitism, as well as between short-term laboratory and long-term laboratory samples. Similarity analysis (ANOSIM) was performed to compare the gut bacteria communities of FAW caterpillars under different treatments. Statistical analysis of the experimental data was conducted using SPSS 23.0, and graphical representations were generated using GraphPad Prism 8.

## 3. Results

### 3.1. Annotation and Evaluation of Gut Bacterial Species in FAW Caterpillars

16S rRNA sequencing was performed on FAW gut samples, resulting in a total of 663,035 sequences. Detailed sequencing information for each sample is presented in Table 1. Subsequent OTU analysis was conducted at a sequence similarity level of 97%, leading to the identification of 10 phyla, 15 classes, 44 orders, 63 families, 80 genera, 93 species, and a total of 99 OTUs.

### 3.2. Gut Microbial Diversity in FAW Caterpillars

**Figure 2:**
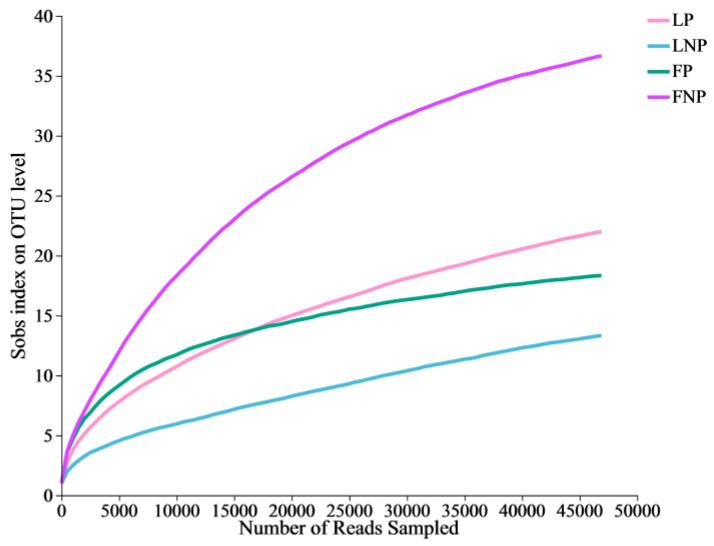
Rarefaction curves of each sample based on Miseq sequencing. The *X*-axis represents the amount of data randomly selected, and the *Y*-axis represents the number of species observed. LP represents the long-term laboratory *Spodoptera frugiperda* parasitized by *Cotesia ruficrus*. LNP represents the long-term laboratory non-parasitized *S. frugiperda*. FP represents the short-term laboratory parasitized *S. frugiperda*. FNP represents the short-term laboratory non-parasitized *S. frugiperda*.

**Figure 3:**
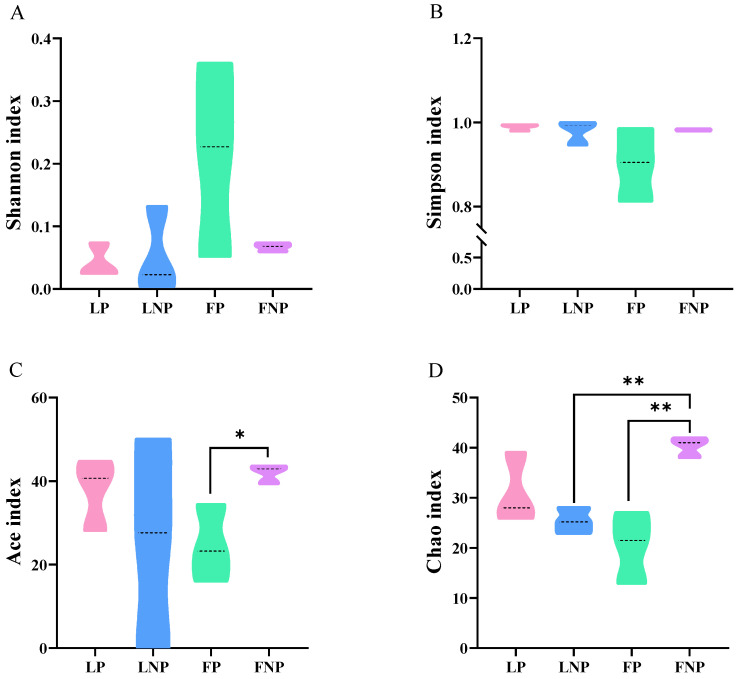
Alpha diversity of gut microbiota in *Spodoptera frugiperda.* (**A**) Shannon index; (**B**) Simpson index; (**C**) Ace index; and (**D**) Chao index. The larger the Shannon index value, the higher the community diversity; the larger the Simpson index value, the lower the community diversity; and the larger the Chao and Ace index values, the higher the community richness. LP represents the long-term laboratory *S. frugiperda* parasitized by *Cotesia ruficrus*. LNP represents the long-term laboratory non-parasitized *S. frugiperda*. FP represents the short-term laboratory parasitized *S. frugiperda*. FNP represents the short-term laboratory non-parasitized *S. frugiperda*. Data were analyzed by Student’s t-test, values represent the means ± SE. *, ** indicated that there were significant differences between parasitized and non-parasitized *S. frugiperda* caterpillars at *p* < 0.05 and *p* < 0.01, respectively.

Alpha diversity analysis was performed on the gut bacteria of the FAWs. The results show that the dilution curves of all the samples exhibited stability, indicating the adequacy of the sequencing data volume (Figure 2). A significant difference was observed in the Ace index (*t* = 3.197, *df* = 4, *P* = 0.033) and the Chao index (*t* = 4.690, *df* = 4, *P* = 0.009) of gut bacteria between FP and FNP FAWs (Figure 3C,D). Additionally, a notable distinction was found in the Chao index of gut bacteria between LNP and FNP FAWs (*t* = 8.097, *df* = 4, *P* = 0.001) (Figure 3D).

**Figure 4:**
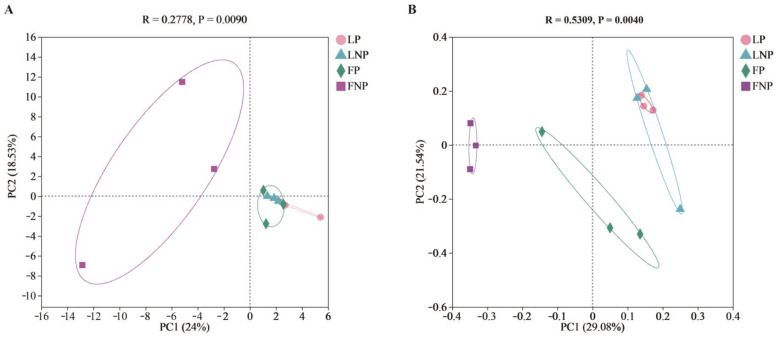
PCA analysis (**A**) and PCoA analysis (**B**) of gut bacterial community in *Spodoptera frugiperda.* LP represents the long-term laboratory *Spodoptera frugiperda* parasitized by *Cotesia ruficrus*. LNP represents the long-term laboratory non-parasitized *S. frugiperda*. FP represents the short-term laboratory parasitized *S. frugiperda*. FNP represents the short-term laboratory non-parasitized *S. frugiperda*. Data were analyzed by ANOSIM.

The PCA analysis indicated a relatively similar bacterial composition among LP, LNP, FP, and FNP, while the similarity analysis revealed significant differences in the gut bacterial communities of FAWs subjected to different treatments (ANOSIM, *R* = 0.2778, *P* = 0.009) (Figure 4A). Furthermore, the PCoA analysis demonstrated a relatively similar bacterial composition between LP and LNP, with discernible differences in the bacterial composition of FP and FNP. The analysis also highlighted significant disparities in the gut bacterial communities of FAWs exposed to different treatments (ANOSIM, *R* = 0.5309, *P* = 0.004) (Figure 4B).

### 3.3. Gut Microbial Composition in FAW Caterpillars

**Figure 5:**
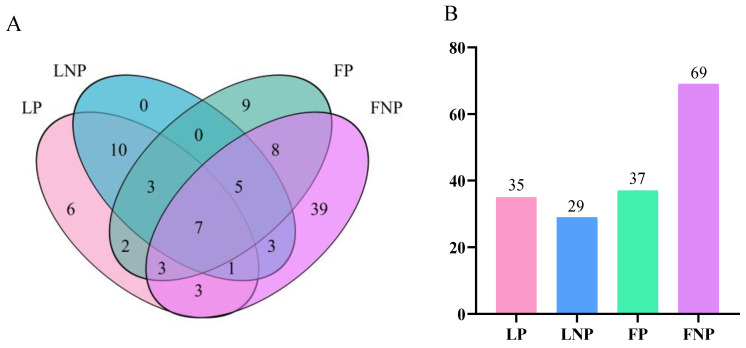
Venn diagram (**A**) and bar chart (**B**) of gut bacterial community in *Spodoptera frugiperda*. LP represents the long-term laboratory *Spodoptera frugiperda* parasitized by *Cotesia ruficrus*. LNP represents the long-term laboratory non-parasitized *S. frugiperda*. FP represents the short-term laboratory parasitized *S. frugiperda*. FNP represents the short-term laboratory non-parasitized *S. frugiperda*.

The Venn diagram illustrating the gut bacterial community composition of FAWs revealed specific patterns: LP and FP shared 2 OTUs, LNP and FNP shared 3 OTUs, LP and LNP shared 10 OTUs, and FP and FNP shared 8 OTUs (Figure 5A). Notably, FNP exhibited the highest number of OTUs, with 69 identified, accounting for 69.70% of the total OTUs (Figure 5B).

**Figure 6:**
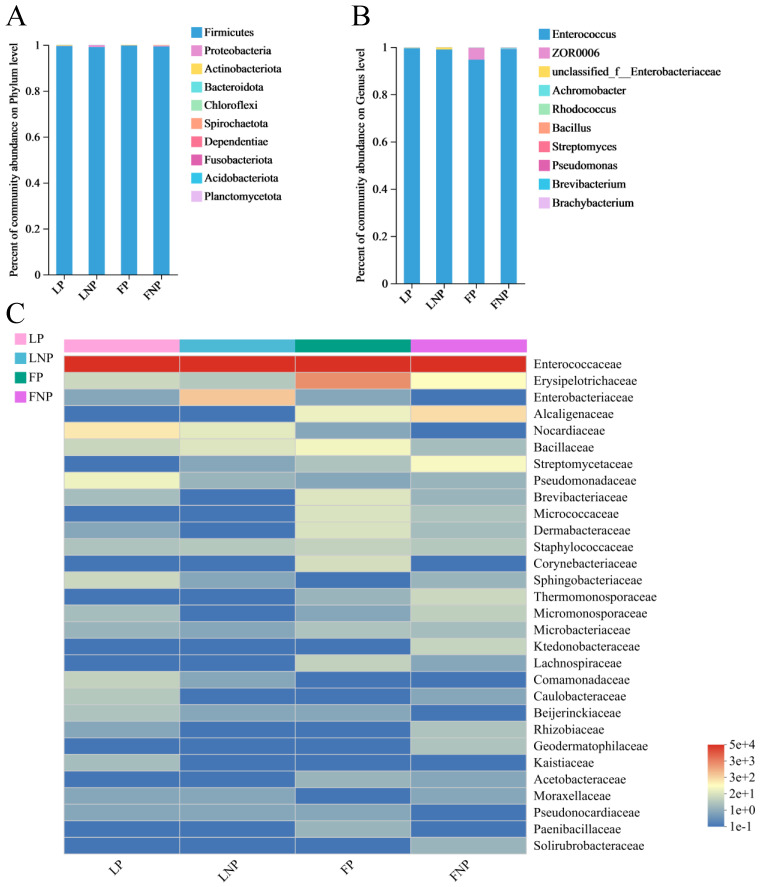
Gut bacterial community dynamics in *Spodoptera frugiperda*. (**A**) Phylum level; (**B**) genus level; and (**C**) family level. LP represents the long-term laboratory *Spodoptera frugiperda* parasitized by *Cotesia ruficrus*. LNP represents the long-term laboratory non-parasitized *S. frugiperda*. FP represents the short-term laboratory parasitized *S. frugiperda*. FNP represents the short-term laboratory non-parasitized *S. frugiperda*.

In the four treatments of LP, LNP, FP, and FNP, Firmicutes emerged as the dominant phylum within the gut bacterial communities at the phylum level, with abundances exceeding 99% across all samples (Figure 6A). Furthermore, at the genus level, *Enterococcus* stood out as the predominant genus, representing over 94% abundance in all samples (Figure 6B). The heatmap depicting the gut bacterial community composition revealed Enterococcaceae as the most abundant family across the four treatments of FAWs. Additionally, Nocardiaceae, Enterobacteriaceae, Erysipelotrichaceae, and Alcaligenaceae emerged as the second most abundant family in LP, LNP, FP, and FNP, respectively. Notably, the abundance of Nocardiaceae exhibited an increase in parasitized FAWs (Figure 6C).

**Figure 7:**
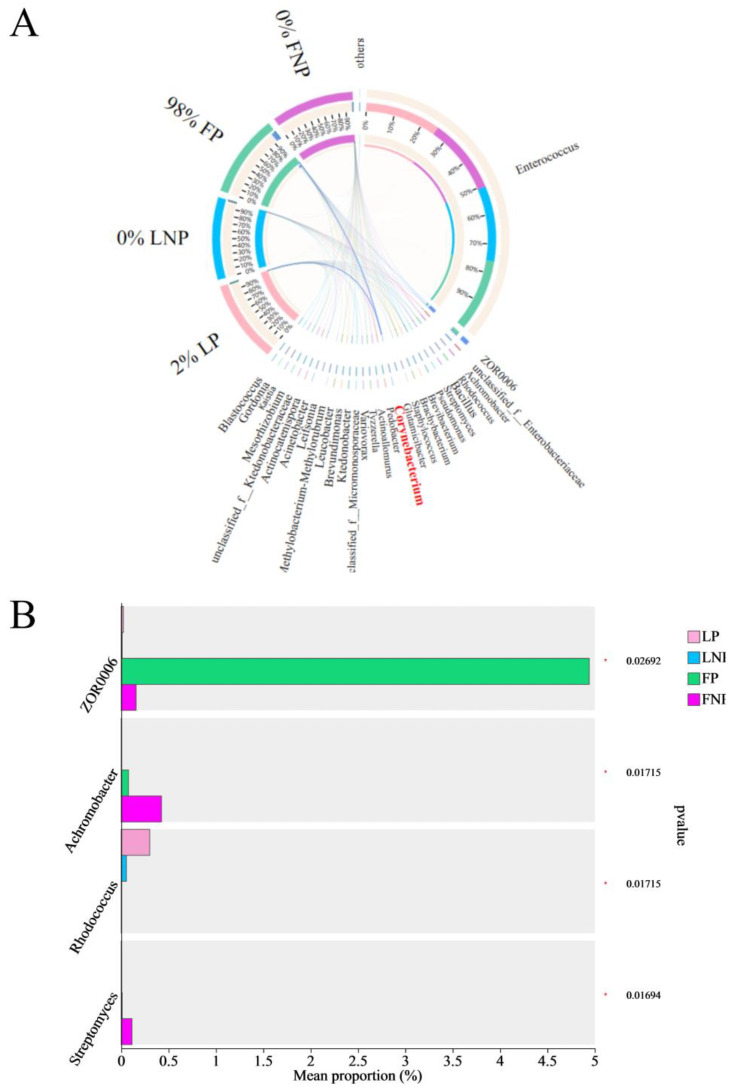
Visual circle graph of gut bacteria (**A**) and gut bacteria significance test (**B**) in *Spodoptera frugiperda*. LP represents the long-term laboratory *Spodoptera frugiperda* parasitized by *Cotesia ruficrus*. LNP represents the long-term laboratory non-parasitized *S. frugiperda*. FP represents the short-term laboratory parasitized *S. frugiperda*. FNP represents the short-term laboratory non-parasitized *S. frugiperda*. Data were analyzed by Kruskal–Wallis test. * indicated that there were significant differences between parasitized and non-parasitized *S. frugiperda* caterpillars at *p* < 0.05 and *p* < 0.01, respectively.

The relative distribution of dominant species within each sample group and the comparative distribution of these dominant species across different groups were visually depicted using a circular graph. Specifically, we represented the abundances of genera with a relative abundance exceeding 30 in the gut bacterial composition of FAWs under LP, LNP, FP, and FNP treatments. Interestingly, our analysis revealed that Corynebacterium was exclusively present in two parasitized FAW caterpillar populations (LP and FP) (Figure 7A). Moreover, ZOR0006, Achromobacter, Rhodococcus, and Streptomyces exhibited significant variations in abundance across the four distinct types of gut bacteria observed in FAWs (Figure 7B).

## 4. Discussion

It is well known that parasitoids play a significant role in regulating various physiological processes of their hosts post-parasitization, including immune response, development, metabolism, and behavior [28,29]. Furthermore, the presence of microorganisms within the insect hosts also contributes to the modulation of these physiological activities [4]. Recently, there has been growing interest in investigating whether parasitoids can influence the gut microbiota of their host insects after parasitism, thereby indirectly impacting the host’s physiological functions. In this study, we used 16S rRNA sequencing technology to examine the effect of the parasitoid *C. ruficrus* on the gut bacterial composition of long-term laboratory and short-term laboratory FAW caterpillar populations. Our findings revealed *Enterococcus* as the predominant bacterium in FAWs. Specifically, for short-term laboratory FAW caterpillars, parasitized individuals exhibited reduced gut microbial richness compared to non-parasitized counterparts, while long-term laboratory rearing led to decreased gut bacterial abundance in FAW caterpillars.

Several studies have highlighted that parasitism by parasitoids can alter the diversity of the host’s gut microbiota [22,30,31]. In this study, Alpha diversity analysis of the gut bacteria of four FAW populations indicated a higher Chao index in short-term laboratory FAWs compared to long-term laboratory FAWs, suggesting greater bacterial richness in short-term laboratory FAWs. This observation aligns with similar findings in various insect species, such as *Triatoma infestans* (Klug) (Hemiptera: Reduviidae) and the fruit fly pest *Anastrepha fraterculus* sp. 1 [32,33]. Moreover, after parasitism by *C. ruficrus*, the Ace index and the Chao index decreased in short-term laboratory FAW populations, indicating a reduction in gut bacterial richness. However, the Shannon index and the Simpson index showed no significant changes, implying that parasitism by *C. ruficrus* did not impact the diversity of gut bacteria in short-term laboratory FAWs. The findings contrast with those of *Cotesia vestalis* (Haliday) (Hymenoptera: Braconidae) parasitizing *Plutella xylostella* (Linnaeus) (Lepidoptera: Plutellidae), which show a decrease in both richness and diversity following parasitism [21]. Conversely, results from *Coccinella septempunctata* Linnaeus (Coleoptera: Coccinellidae) parasitized by *Homalotylus eytelweinii* (Ratzeburg) (Hymenoptera: Encyrtidae) demonstrated an increase in both richness and diversity [21]. Notably, our results indicated no significant differences in the diversity and richness of gut bacteria in long-term laboratory FAWs post-parasitism, potentially due to the low number of OTUs sequenced in long-term indoor-reared FAWs.

Classification analysis identified *Enterococcus*, a member of Firmicutes, as the predominant gut bacteria in FAWs, consistent with its prevalence in other lepidopteran insects like *Spodoptera litura* (Fabricius) (Lepidoptera: Noctuidae) [34], *Helicoverpa armigera* (Hübner) (Lepidoptera: Noctuidae) [35], and *P. xylostella* [36]. *Enterococcus* is known to play crucial roles in insect gut ecology, displaying resilience to plant-secreted latex, alkaloid degradation capabilities, pathogen resistance, metamorphosis regulation, and potential involvement in host insect energy metabolism [37,38,39,40]. Additionally, we found the presence of *Corynebacterium* in the gut of FAWs parasitized by *C. ruficrus*, which can also degrade alkaloids [41]. However, *Corynebacterium* was not detected in non-parasitized FAWs, suggesting two plausible explanations: either *Corynebacterium* exists in non-parasitized FAWs at extremely low levels undetectable by our sequencing method, or it is introduced by *C. ruficrus* during parasitism. Checking if *Corynebacterium* is present in *C. ruficrus* could provide better insights, prompting the need for further experimental investigation. In addition, we found that ZOR0006 is the dominant bacteria in short-term laboratory parasitized FAWs. Studies have reported that ZOR0006 may cause changes in the host’s metabolites [42]. In our previous research, we found that the metabolites of FAW caterpillars changed after being parasitized by *C. ruficrus*. Whether ZOR0006 is involved in this process requires further study.

The gut microbiota composition of insects undergoes variations across different developmental stages. While our study focused on gut bacteria sequencing in FAWs 5 days post-parasitism by *C. ruficrus*, temporal changes in FAW gut microbiota post-parasitism warrant further exploration. Furthermore, the regulatory role of parasitic factors, such as venom [43], teratocytes [44], and polydnaviruses on host immunity [45,46], development, metabolism, and behavior post-parasitism are well documented. Therefore, it is conceivable that alterations in host insect gut microbiota post-parasitism may also be influenced by parasitic factors. This hypothesis finds support in instances like polydnaviruses from *C. vestalis* altering the gut microbiota of *P. xylostella* [39]. Our study identified significant changes in the gut microbes of FAWs following parasitization by *C. ruficrus*, indicating a potential association between these alterations and parasitic factors. This discovery underscores the need for further investigation. In addition, since gut microorganisms regulate insects’ immunity, development, and metabolism, after parasitoids parasitize host insects, changing the composition of the host’s gut microbiota may alter the host’s nutritional metabolism and immune response, thus benefiting the survival of the parasitoid larvae. For example, the larvae of *Leptopilina boulardi* (Barbotin, Carton and Keiner-Pillault) (Hymenoptera: Figitidae) rely on the gut microbiota of their host *D. melanogaster* to increase the host’s lipid storage, thereby satisfying the nutritional requirements and survival of the parasitoid wasp larvae [47]. After the *C. ruficrus* parasitized FAWs, it changed the gut microbial structure of FAW caterpillars. Whether the change in microorganisms further affects the physiological processes of FAW caterpillars requires further exploration.

## 5. Conclusions

In summary, our study reveals that *C. ruficrus* parasitism induces changes in FAW larval gut microbiota, shedding light on the capacity of parasitoid parasitism to impact host gut microbiota. These findings lay a foundation for deeper exploration of the gut microbiota’s role in the parasitoid–host interaction mechanism.

## Figures and Tables

**Table 1:** 16srRNA sequencing information of *Spodoptera frugiperda*.

Sample	Seq-Number	Base-Number	Mean-Length	Min-Length	Max-Length
LP1	52,551	22,543,489	428.9831	262	433
LP2	47,536	20,390,468	428.9479	266	431
LP3	55,737	23,898,913	428.78	371	446
LNP1	54,045	23,180,439	428.91	262	431
LNP2	47,744	20,479,500	428.944	404	434
LNP3	53,151	22,799,333	428.954	381	431
FP1	56,372	24,173,536	428.8217	381	430
FP2	58,708	25,152,659	428.4367	277	519
FP3	57,021	24,455,259	428.8816	270	431
FNP1	59,528	25,520,352	428.7117	262	431
FNP2	64,040	27,427,768	428.2912	374	518
FNP3	56,602	24,267,829	428.7451	262	518

Note: LP1, LP2, and LP3 represent three replicates of the long-term laboratory *Spodoptera frugiperda* parasitized by *Cotesia ruficrus*. LNP1, LNP2, and LNP3 represent three replicates of the long-term laboratory non-parasitized *S. frugiperda*. FP1, FP 2, and FP 3 represent the short-term laboratory parasitized *S. frugiperda*. FNP1, FNP2, and FNP3 represent the short-term laboratory non-parasitized *S. frugiperda*.

## Data Availability

Raw sequencing data were deposited in the NCBI Short Read Archive (SRA) BioProject PRJNA1097026.
